# Impact of a Single Point Mutation on the Antimicrobial and Fibrillogenic Properties of Cryptides from Human Apolipoprotein B

**DOI:** 10.3390/ph14070631

**Published:** 2021-06-29

**Authors:** Rosa Gaglione, Giovanni Smaldone, Angela Cesaro, Mariano Rumolo, Maria De Luca, Rocco Di Girolamo, Luigi Petraccone, Pompea Del Vecchio, Rosario Oliva, Eugenio Notomista, Emilia Pedone, Angela Arciello

**Affiliations:** 1 Department of Chemical Sciences, University of Naples Federico II, 80126 Naples, Italy; rosa.gaglione@unina.it (R.G.); angela.cesaro@unina.it (A.C.); marianorumolo@gmail.com (M.R.); maria.deluca2@unina.it (M.D.L.); rocco.digirolamo@unina.it (R.D.G.); luigi.petraccone@unina.it (L.P.); pompea.delvecchio@unina.it (P.D.V.); 2 Istituto Nazionale di Biostrutture e Biosistemi (INBB), 00136 Rome, Italy; 3 IRCCS SDN, Via E. Gianturco 113, 80143 Naples, Italy; giovanni.smaldone@synlab.it; 4 Physical Chemistry I—Biophysical Chemistry, Faculty of Chemistry and Chemical Biology, TU Dortmund University, 44227 Dortmund, Germany; rosario.oliva@tu-dortmund.de; 5 Department of Biology, University of Naples Federico II, 80126 Naples, Italy; notomist@unina.it; 6 Istituto di Biostrutture e Bioimmagini, CNR, 80134 Naples, Italy; empedone@unina.it; 7 Research Centre on Bioactive Peptides (CIRPeB), University of Naples Federico II, Via Mezzocannone 16, 80134 Naples, Italy

**Keywords:** bioactive cryptides, single point mutation, anti-biofilm activity, in vitro fibrillogenesis

## Abstract

Host defense peptides (HDPs) are gaining increasing interest, since they are endowed with multiple activities, are often effective on multidrug resistant bacteria and do not generally lead to the development of resistance phenotypes. Cryptic HDPs have been recently identified in human apolipoprotein B and found to be endowed with a broad-spectrum antimicrobial activity, with anti-biofilm, wound healing and immunomodulatory properties, and with the ability to synergistically act in combination with conventional antibiotics, while being not toxic for eukaryotic cells. Here, a multidisciplinary approach was used, including time killing curves, differential scanning calorimetry, circular dichroism, ThT binding assays, and transmission electron microscopy analyses. The effects of a single point mutation (Pro → Ala in position 7) on the biological properties of ApoB-derived peptide r(P)ApoB_L_^Pro^ have been evaluated. Although the two versions of the peptide share similar antimicrobial and anti-biofilm properties, only r(P)ApoB_L_^Ala^ peptide was found to exert bactericidal effects. Interestingly, antimicrobial activity of both peptide versions appears to be dependent from their interaction with specific components of bacterial surfaces, such as LPS or LTA, which induce peptides to form β-sheet-rich amyloid-like structures. Altogether, obtained data indicate a correlation between ApoB-derived peptides self-assembling state and their antibacterial activity.

## 1. Introduction

Bacterial resistance to commonly used drugs, which is causing a huge increase in infection cases and mortality rate, has become a great public concern in the 21st century [1,2]. Furthermore, one of the main virulence determinants in many bacterial infections is biofilm formation, which significantly increases bacterial resistance to conventional antibiotics and innate host defense [3]. In this scenario, the search for alternative effective agents able to counteract chronic infections and to reduce the ability of pathogens to form biofilms is becoming urgent. Among available alternatives, host defense peptides (HDPs), frequently referred to as antimicrobial peptides (AMPs), have attracted considerable attention because of their wide range of biological activities, including antimicrobial, anti-biofilm, anticancer, immunomodulatory, and wound healing properties [4]. They are innate immune system effectors produced by all complex animals, insects and plants, and generally have modest direct activity against a broad range of microorganisms including bacteria, viruses, fungi, and protozoa. Interestingly, their capability to kill microbes through multiple mechanisms makes the development of bacterial resistance unlikely [5,6,7,8,9,10,11,12,13]. The mechanism of action of HDPs is generally based on membrane binding and membrane destabilization as common essential steps for them to exert their antimicrobial properties [14]. Recently, a novel tassel has been added to the picture describing the molecular events at the basis of HDPs mechanism of action. Indeed, it has been reported that HDPs, such as bacteriocins and temporins, or proteins like lysozyme, lactoferrin, eosinophil, and cationic proteins, are able to form amyloid-like structures that play a key role in bacterial membranes destabilization [15,16,17,18,19]. Although the molecular mechanisms by which amyloid peptides exert their antimicrobial activity has not been fully elucidated yet, collected experimental evidences indicate channel formation as a common mechanism of action amongst these amyloidogenic peptides [20,21,22,23,24,25,26], thus indicating a correlation between amyloid propensity and antimicrobial activity. Interestingly, recent data also support a key physiological antimicrobial role for Aβ peptide, responsible for the formation of amyloid plaques in the brain of Alzheimer disease patients [27]. Indeed, microbial DNA has been identified within Aβ plaques [28,29], thus suggesting that Aβ peptide might aggregate in response to the presence of infectious agents in the brain. Moreover, the analysis of three Alzheimer Disease brains, characterized by an infection associated to the spirochete *Borrelia burgdorferi*, revealed that Aβ plaques co-localize with bacterial antigens and DNA [30]. Analyses conducted in vitro also revealed that the treatment of neurons with the factor *Tat* of human immunodeficiency virus (HIV) causes an increase of soluble Aβ concentration and a reduced activity of the Aβ-degrading enzyme neprilysin, thus indicating that neurons respond to the presence of HIV by upregulating Aβ expression [31,32]. Several recent studies also describe that amyloidogenic Aβ_40_ and Aβ_42_ peptides, responsible for the formation of plaques in the brain of Alzheimer disease patients, exert direct antimicrobial effects on a variety of human pathogens [33], thus supporting the primary antimicrobial role of the pathogenic peptides and the ability of amyloid-like structures to destabilize bacterial membranes.

It has been reported that many proteins, whose functions are not necessarily related to host defense, can act as sources of HDPs [34,35]. Indeed, the term “cryptome” has been used to define the collection of precursor proteins able to generate bioactive peptides upon proteolytic events [36]. This is also the case of apolipoproteins, proteins commonly known for their role in lipid transport into the blood, such as apolipoprotein B and E that have been found to represent a precious source of cryptic HDPs [37,38,39,40]. Peptides hidden within the sequences of precursor proteins have been defined “cryptides” and have been found to be involved in a wide range of processes including neuronal signalling, antigen presentation, and inflammatory response [36,41,42,43,44].

Recently, our research group identified and characterized a cryptide hidden in human apolipoprotein B. Two versions of the identified peptide, named r(P)ApoB_L_^Pro^ and r(P)ApoB_S_ ^Pro^, have been recombinantly produced and characterized [9,10,11,37,45,46]. L and S indicate a longer or a shorter version of the identified sequence, respectively, and P refers to a Pro residue present at the N-terminal extremity of recombinant peptides because of the excision method used where peptides are released after the acidic cleavage of an Asp-Pro bond [47]. Recombinant ApoB-derived cryptides have been found to be endowed with a broad-range antimicrobial activity, being effective on both Gram-negative and Gram-positive bacterial strains, with a notable anti-biofilm activity and with the ability to synergistically act in combination with conventional antibiotics [9]. Moreover, peptides have been found to exhibit immunomodulatory properties, to target sensitive bacteria by depolarizing their cytoplasmic membrane, to display excellent cytotoxicity profiles, and not to select for bacterial resistance mechanisms [9,10,11,37,48,49].

The main goal of the present study is to evaluate the effects of a single point mutation on the biological properties of r(P)ApoB_L_ peptide. In particular, the Pro residue present in position 887 of a fully functional ApoB isoform and in position 7 of recombinant r(P)ApoB_L_ peptide [9] has been substituted with the Ala residue normally present in the same position in the most abundant ApoB isoform. By means of a multidisciplinary approach including biochemical, microbiological, spectroscopic, and electron microscopy techniques, the effects of the single point mutation on peptides antimicrobial and anti-biofilm properties have been evaluated. Conformational analyses have been also performed upon peptides incubation with key components of bacterial membrane and cell wall, such as lipopolysaccharide (LPS) or lipoteichoic acid (LTA), and the role of induced β-sheet-rich oligomeric structures in antimicrobial activity has been investigated.

## 2. Results

### 2.1. Design of Pro → Ala Mutation in Position 7 of r(P)ApoB_L_ Peptide

The main aim of the present study is to evaluate the effects of an amino acid substitution in position 7 of r(P)ApoB_L_ antimicrobial peptide. Indeed, by analysing the ApoB protein sequence identified as a putative antimicrobial region (amino acids 882–929), it was found that Pro is the residue present in position 887 of a fully functional ApoB isoform [9], whereas Ala is the amino acid residue normally present in the most abundant ApoB isoform. Based on this, we decided to analyse both the versions of r(P)ApoB_L_ peptide. To obtain the version of the peptide with Ala in position 7 instead of Pro, we performed a site-directed mutagenesis experiment by QuickChange site-directed mutagenesis kit [11] and recombinantly produced both the versions of the peptide, here named r(P)ApoB_L_^Pro^ and r(P)ApoB_L_^Ala^. Peptides’ purity and integrity were evaluated by gel-electrophoresis and mass spectra analyses [46]. Sequences and physico-chemical properties of the two versions of the peptide are reported in Table 1.

### 2.2. Evaluation of the Effects of Pro → Ala Single Point Mutation on the Antimicrobial Activity of r(P)ApoB_L_ Peptide

To evaluate whether Pro → Ala substitution in position 7 of recombinant r(P)ApoB_L_ affects peptide antimicrobial activity, both versions of the peptide were tested on a panel of Gram-negative and Gram-positive bacterial strains (Table 2). On the basis of determined MIC_100_ values, r(P)ApoB_L_^Ala^ peptide was found to be endowed with a broad-range antimicrobial activity, exerting significant antimicrobial effects on all the bacterial strains tested. Being its antimicrobial activity comparable to that of r(P)ApoB_L_^Pro^ peptide, it can be concluded that Pro → Ala substitution in position 7 does not significantly affect the peptide antimicrobial properties.

### 2.3. Evaluation of the Effects of Pro → Ala Substitution on the Anti-Biofilm Properties of r(P)ApoB_L_ Peptide

**Figure 1:**
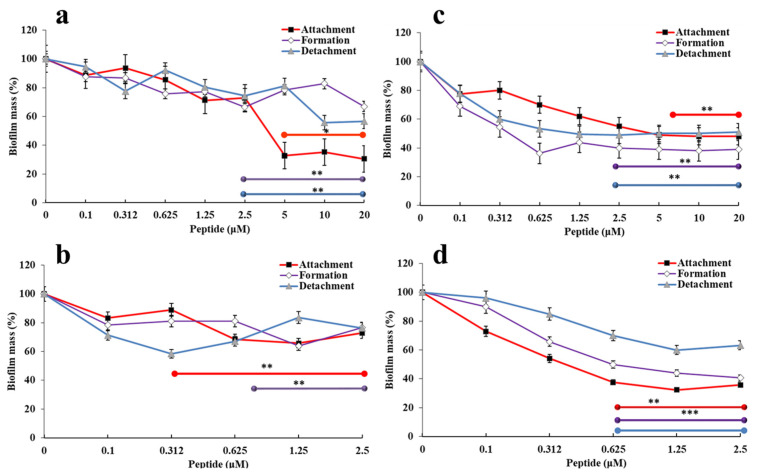
Anti-biofilm activity of r(P)ApoB_L_^Ala^ (**a**,**b**) and r(P)ApoB_L_^Pro^ (**c**,**d**) on *P. aeruginosa* PAO1 (**a**,**c**) and *S. aureus* MRSA WKZ-2 (**b**,**d**) biofilms. The effects of increasing concentrations of the peptide were evaluated on biofilm attachment (black rectangles), biofilm formation (white rhombi) and detachment (grey triangles). Data represent the mean (±standard deviation, SD) of at least three independent experiments, each one carried out with triplicate determinations. * *p* < 0.05, ** *p* < 0.01 and *** *p* < 0.001 were obtained for control *versus* treated samples.

To compare the anti-biofilm properties of the two versions of the peptide, crystal violet (CV) staining assays were performed to test the effects of increasing concentrations (0–20 µM) of r(P)ApoB_L_^Ala^ and r(P)ApoB_L_^Pro^ peptides on the three main stages of biofilm development, such as biofilm attachment, formation and detachment (Figure 1).

To this purpose, following incubation with peptides, biofilm samples were analysed by crystal violet staining that revealed a significant dose-dependent inhibition of biofilm attachment and formation in the case of both *P. aeruginosa* (Figure 1a,c) and *S. aureus* MRSA WKZ-2 (Figure 1b,d) upon treatment with both peptides, that were found to display a significant anti-biofilm activity even at peptide concentrations significantly lower than those required to directly kill planktonic cells (0.1–10 µM in the case of *P. aeruginosa* PAO1 and 0.1–1.25 µM in the case of *S. aureus* MRSA WKZ-2). In the case of biofilm detachment, peptides were found to be able to significantly eradicate biofilm only in the case of *P. aeruginosa.* As shown in Figure 1, the results obtained in the case of r(P)ApoB_L_^Ala^ peptide are similar to those obtained and previously described for r(P)ApoB_L_^Pro^ [9], thus indicating that Pro → Ala substitution doesn’t significantly affect peptide anti-biofilm properties.

### 2.4. Evaluation of the Effects of Pro → Ala Single Point Mutation on the Kinetics of Bacterial Killing

**Figure 2:**
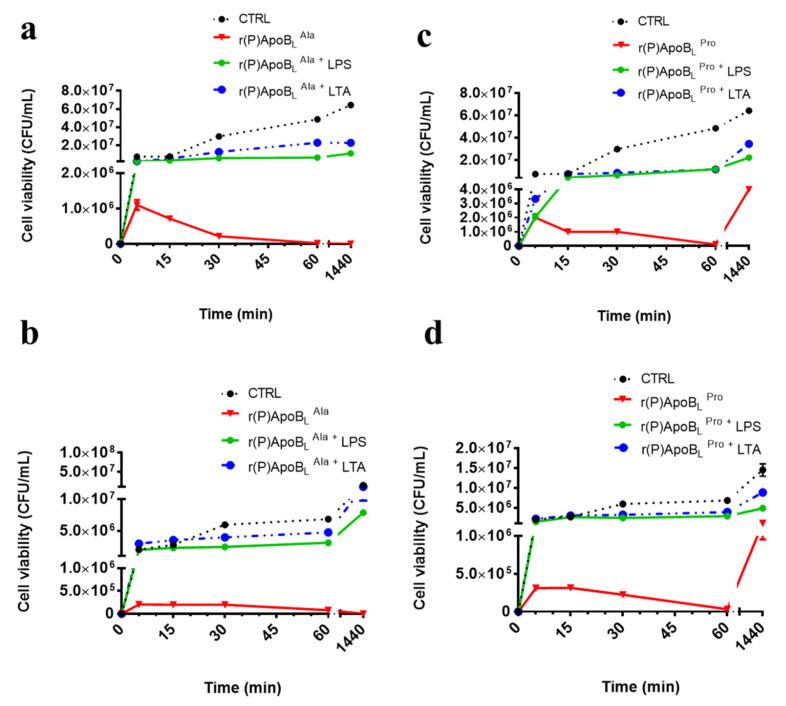
Time killing curves obtained upon incubation of *P. aeruginosa* PAO1 (**a**,**c**) and *S. aureus* MRSA WKZ-2 (**b**,**d**) bacterial strains with either r(P)ApoB_L_^Ala^ or r(P)ApoB_L_^Pro^ alone or upon pre-incubation with LPS or LTA. Experiments have been performed two times with intra-experimental triplicates.

To verify whether Pro → Ala substitution has effects on the kinetic of peptides bactericidal activity, kinetic killing curves were obtained upon treatment of *P. aeruginosa* PAO1 and *S. aureus* MRSA WKZ-2 bacterial cells with each peptide for different time intervals (0–24 h) (Figure 2). 

These two bacterial strains have been selected as prototypes of Gram-positive (*S. aureus* MRSA WKZ-2) and Gram-negative (*P. aeruginosa* PAO1) strains susceptible to peptide antimicrobial activity (Table 2 and Figure 2). In all the experiments, peptides were tested at a concentration of 10 μM. As reported in Figure 2a,b, the peptides are able to exert toxic effects on both strains within 60 min, but only r(P)ApoB_L_^Ala^ is able to exert a bactericidal action, since no colonies are detected upon 24 h incubation (Figure 2a,b). In the case of r(P)ApoB_L_^Pro^ peptide, instead, the detection of colonies upon 24 h incubation is indicative of the persistence of viable bacterial cells under the experimental conditions tested (Figure 2c,d).

Based on the hypothesis that peptides’ antimicrobial activity might be mediated by the interaction with key components of Gram-positive bacteria cell wall or Gram-negative bacteria outer membrane, time killing experiments were also performed upon a pre-incubation of the peptides with the endotoxin LPS, the predominant glycolipid in the outer membrane of Gram-negative bacteria [50], or with LTA, an important component of Gram-positive bacteria cell wall. LPS and LTA were tested at a concentration of 0.1 mg/mL for 15 min at 37 °C and analyses were performed as previously described [51]. As reported in Figure 2, when peptides have been pre-incubated with LPS or LTA, their antimicrobial activity appears significantly reduced probably because free LPS and LTA compete with molecules exposed on bacterial surfaces for the binding to peptide molecules. This strongly suggests that the interaction of ApoB-derived peptides with LPS or LTA is crucial for peptides’ antimicrobial activity.

### 2.5. Evaluation of the Effects of Pro → Ala Single Point Mutation on Peptide Interaction with Double Lipid Layer by Differential Scanning Calorimetry (DSC) Analyses

**Figure 3:**
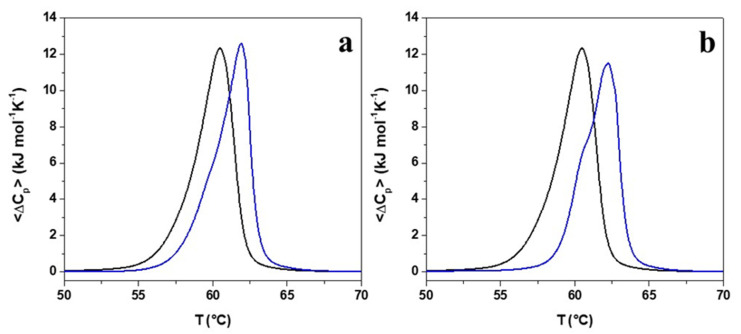
DSC thermograms of DPPE/DPPG in the absence (black lines) and in the presence of (**a**) r(P)ApoB_L_^Pro^ and (**b**) r(P)ApoB_L_^Ala^ at L/P molar ratio of 10 (blue lines). All the experiments were performed in 20 mM Phosphate buffer, pH 7.4.

To analyse the role played by peptides’ interaction with lipid bilayer in the mechanism underlying peptides’ antimicrobial activity and to evaluate whether Pro → Ala single point mutation affects peptide interaction with the lipid bilayer, differential scanning calorimetry (DSC) experiments were performed. To this purpose, liposomes composed of dipalmitoyl phosphatidylethanolamine/1,2-dipalmitoyl-sn-glycero-3-phosphoglycerol (DPPE/DPPG) 8/2 (mol/mol) were used as a simple model simulating bacterial membranes. In particular, 0.5 mM of preformed DPPE/DPPG multilamellar vesicles (MLVs) were mixed with either r(P)ApoB_L_^Pro^ or r(P)ApoB_L_^Ala^ at lipid-to-peptide (L/P) molar ratio of 10 (mol/mol). The obtained thermograms are reported in Figure 3a,b. As it is shown, the DSC thermogram of the DPPE/DPPG mixture in the absence of the peptide is centred at 60.5 °C and the gel-to-liquid phase transition is characterized by the appearance of a single broad peak, thus indicating that the two lipids are miscible [52]. Upon peptides addition, the DPPE/DPPG thermogram drastically changes revealing a strong interaction of the peptides with the lipid bilayer. For both peptides, a similar increase of the melting temperature of the DPPE/DPPG gel-to-liquid transition was observed (Table 3). Furthermore, the DSC profiles show a shoulder at about 60 °C (more evident in the case of r(P)ApoB_L_^Ala^), indicative of the presence of two separate transitions, thus suggesting the formation of lipid domains upon peptides binding, in agreement with data previously reported for different antimicrobial peptides [53,54,55]. This behavior could be due to the partial segregation of the DPPG lipid in the DPPE/DPPG membrane as a result of a preferential interaction of the positively charged peptide molecules with the negatively charged DPPG lipids. DPPG segregation consequently leads to the formation of domains with significantly higher DPPE content that melt at higher temperature values (pure DPPE melts at 63 °C). Moreover, as reported in Table 3, the enthalpy change of the overall gel-to-liquid phase transition is not drastically affected by the presence of peptides, revealing that peptides are not able to destabilize the hydrophobic core of the lipid bilayer. This observation, together with the increase of the gel-to-liquid transition temperature, is consistent with a surface localization of the peptides [54,56]. Although the peptides’ behaviors are qualitatively similar, a detailed inspection of Table 3 shows that the addition of r(P)ApoB_L_^Ala^ results in a higher temperature shift and in a greater decrease of enthalpy changes when compared with r(P)ApoB_L_^Pro^, thus suggesting that Pro → Ala substitution slightly improves the membrane-perturbing capability of the peptide, possibly due to an increase of the peptide hydrophobicity.

### 2.6. Evaluation of the Effects of Pro → Ala Single Point Mutation on r(P)ApoB_L_ Conformation by Far-UV Circular Dichroism Analyses

**Figure 4:**
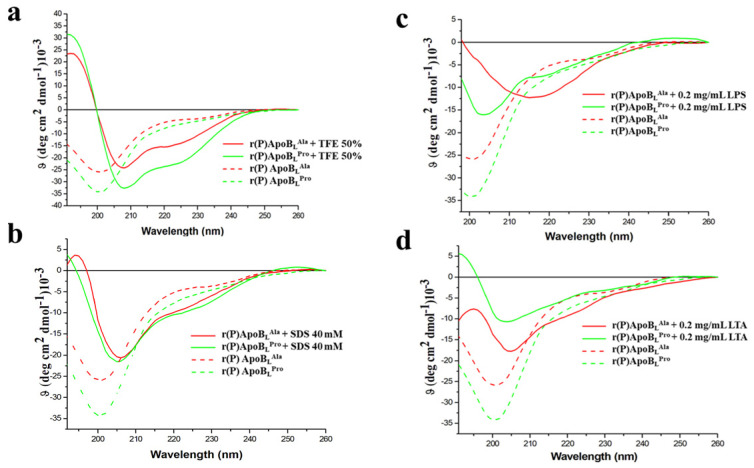
Far-UV CD spectra of r(P)ApoB_L_^Pro^ and r(P)ApoB_L_^Ala^ in the presence of TFE (**a**), SDS (**b**), LPS (**c**) or LTA (**d**). Dashed and dotted lines represent peptides in 20 mM Sodium Phosphate at pH 7.4.

To deepen on the role played by peptides interaction with bacterial membranes or with bacterial surface components in the molecular mechanism underlying peptides’ antimicrobial activity, peptides’ conformations were analysed in the presence of membrane-mimicking agents or in the presence of key components of Gram-negative outer membrane and Gram-positive cell wall, such as LPS or LTA. To this purpose, Far-UV circular dichroism analyses were performed. The secondary structure of recombinant r(P)ApoB_L_^Ala^ peptide was found to be largely unstructured in 20 mM sodium phosphate pH 7.4 buffer (Figure 4a,b), in agreement with data previously reported for r(P)ApoB_L_^Pro^ peptide [9]. The effects of membrane-mimicking agents, such as TFE and SDS at micellar concentrations, on peptide conformation were also evaluated. r(P)ApoB_L_^Ala^ secondary structure was found to shift towards an α-helical conformation in the presence of both membrane-mimicking agents (Figure 4a,b; Supplementary Figure S1a,b). This is clearly evidenced by the presence of two broad minima at around 208 and 222 nm, and a maximum at <200 nm (Figure 4a,b). This observation was confirmed by CD spectra deconvolution data that indicated a shift from a mainly unstructured to a mainly α-helical conformation (Supplementary Tables S1 and S2). A similar behavior has been previously described for r(P)ApoB_L_^Pro^ peptide [9], thus indicating that both peptides are prone to assume a specific ordered conformation when interacting with membrane-mimicking agents.

We also analysed by CD spectroscopy the effects of the endotoxin LPS, the predominant glycolipid in the outer membrane of Gram-negative bacteria [50], on r(P)ApoB_L_^Ala^ peptide conformation (Figure 4c and Supplementary Figure S2). In the presence of increasing concentrations (from 0.1 to 0.8 mg/mL) of *E. coli* LPS, the immediate appearance of a maximum at approximately 200 nm and of a minimum at approximately 218 nm suggests that the peptide tends to assume a prevalently β-strand conformation, probably induced by its interaction with LPS (Figure 4c). Similar results have been previously described for r(P)ApoB_L_^Pro^ peptide, which was found to progressively assume a β-strand conformation when incubated with LPS increasing concentrations, with an almost complete conformational transition in the presence of 0.6 mg/mL LPS [9]. In the case of r(P)ApoB_L_^Ala^ peptide, a similar effect was obtained in the presence of significantly lower LPS concentrations (0.1 mg/mL), thus suggesting that Pro → Ala substitution is responsible for a higher affinity of the peptide for LPS. Being LTA an important component of Gram-positive bacteria cell wall, we also analysed for the first time the effects of this compound on r(P)ApoB_L_^Pro^ and r(P)ApoB_L_^Ala^ peptides conformation (Figure 4d). Because of background noise, LTA was tested only at a concentration of 0.2 mg/mL. In the case of both peptides, a significant decrease of intensity signal and a shift of the minimum indicative of a transition towards a β-strand conformation has been observed, thus clearly indicating that both peptides undergo similar conformational transitions upon incubation with LTA. The secondary structure transition has been also confirmed by CD spectra deconvolution analyses (Supplementary Tables S3 and S4). Altogether, obtained data confirm that both peptides are able to interact with LPS and LTA, even if with different affinity, and, on the basis of time killing curves analyses described above, this interaction appears to play a key role in peptides’ antimicrobial activity.

### 2.7. Conformational Analyses of r(P)ApoB_L_^Pro^ or r(P)ApoB_L_^Ala^ Peptides in the Presence of Susceptible Bacterial Cells

**Figure 5:**
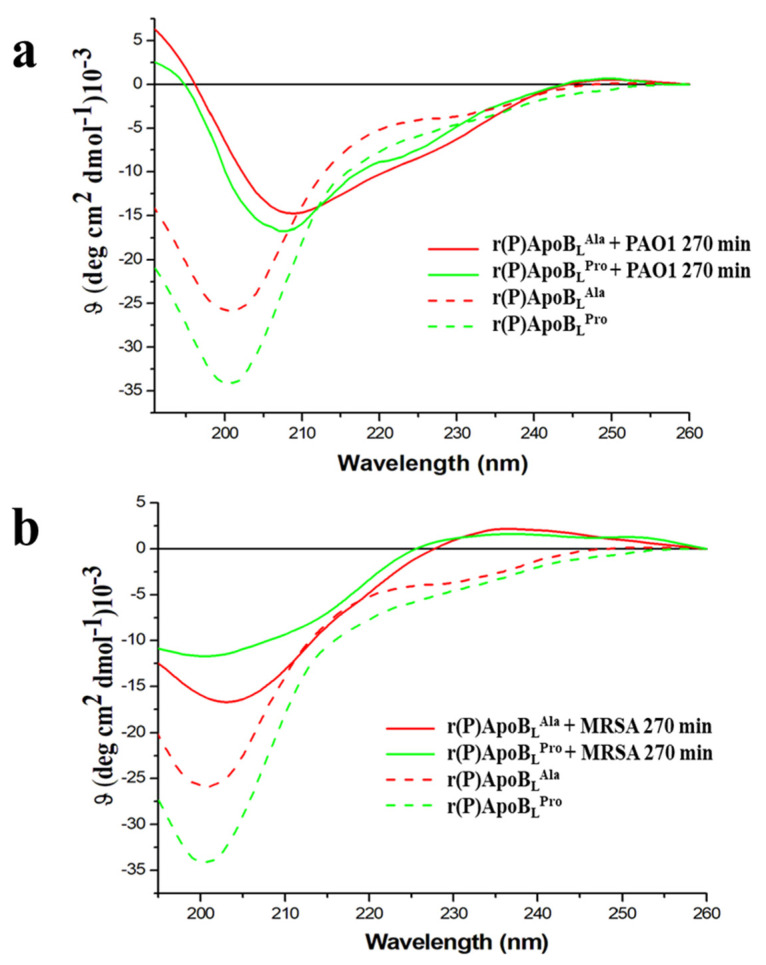
Far-UV CD spectra of r(P)ApoB_L_^Pro^ and r(P)ApoB_L_^Ala^ in the presence of Gram-negative *P. aeruginosa* PAO1 (**a**) or in the presence of Gram-positive *S. aureus* MRSA WKZ-2 (**b**). Dashed lines represent peptides in 20 mM sodium phosphate pH 7.4.

To confirm that the interaction between ApoB-derived peptides and bacterial surfaces is crucial for peptides to exert antimicrobial activity, peptides’ conformational analyses were performed upon incubation with susceptible bacterial cells by circular dichroism spectroscopy. Gram-negative *P. aeruginosa* PAO1 and Gram-positive *S. aureus* MRSA WKZ-2 were selected as prototypes of Gram-negative and Gram-positive bacterial strains responsive to ApoB-derived peptides’ antimicrobial activity. To perform the analyses, we employed an experimental procedure similar to that reported in previously published papers [57,58,59]. Each peptide was incubated at a concentration of 10 μM in the presence of bacterial cells diluted at 0.5 OD_600 nm_/mL in 20 mM sodium phosphate pH 7.4 for 270 min, and CD spectra were recorded at regular time intervals. The CD bacterial cells spectra were subtracted from those of the peptides in the presence of the cells (Supplementary Figure S3). As shown in Figure 5a,b and in Supplementary Figures S4 and S5, a progressive transition from a prevalently random-coil conformation to a more structured conformation was observed. Conformation shifts from random coil to β-sheet, indeed, were observed in the presence of *S. aureus* MRSA WKZ-2 after 270 min of incubation (Figure 5b), and the effect was found to be even more pronounced in the case of r(P)ApoB_L_^Pro^, thus suggesting peptide interaction with specific exposed cell components. In the case of *P. aeruginosa* PAO1 bacterial cells, instead, a slight transition from random coil to α-helix was observed (Figure 5a). Furthermore, since signal intensity was found to decrease over time, it is possible to hypothesize that the interaction between peptide molecules and cell surface is followed by a progressive peptide internalization.

### 2.8. ApoB-Derived Peptides Self-Assembly

Since circular dichroism spectroscopy data indicate that both ApoB-derived peptides shift from an unstructured to a β-strand conformation upon incubation with LPS or LTA (Figure 4c,d), it has been hypothesized that not only this interaction might play a crucial role in the molecular mechanism underlying peptides’ antimicrobial activity, but also that, upon such interaction, peptides might form β-sheet-rich amyloid-like fibrils responsible for their toxic effects. Indeed, in recent years, several papers highlight the antimicrobial properties of amyloids and some human antimicrobial peptides have been found to kill microbes by a channel-forming mechanism based on the formation of extended amyloid fibrils very similar to those of classic disease-forming amyloids [23,60].

**Figure 6:**
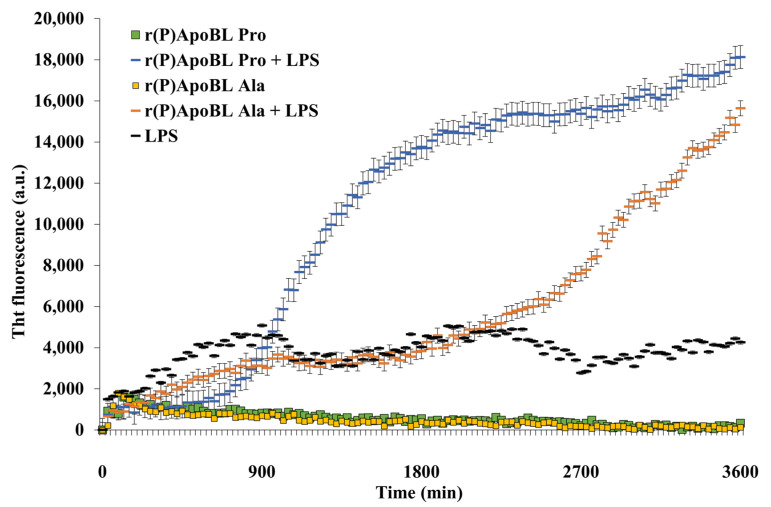
In situ real-time ThT fluorescence assays of r(P)ApoB_L_^Pro^ and r(P)ApoB_L_^Ala^ peptides upon incubation with LPS. In all the cases, reported data derive from three independent experiments with internal triplicate determinations.

The ability of ApoB-derived peptides to form amyloid fibrils has been assessed by in vitro assays. Firstly, ThT fluorescent probe, able to selectively bind to β-sheet-rich amyloid aggregates with a consequent shift of fluorescence emission, has been used to monitor over time r(P)ApoB_L_^Pro^ and r(P)ApoB_L_^Ala^ aggregation into fibrils in the presence of LPS or LTA. The incubation of each peptide (10 µM) with LPS or LTA (0.1 mg/mL) was performed in 20 mM sodium phosphate pH 7.4 for 3 days. A progressive increase of ThT fluorescence emission, indicative of its binding to β-sheet-rich amyloid aggregates, was observed in the case of both versions of the peptide upon incubation with LPS. Increase in fluorescence emission was found to be significantly higher in the case of r(P)ApoB_L_^Pro^ peptide (Figure 6). When peptides were incubated with LTA, no increase in ThT fluorescence emission was instead detected (Supplementary Figure S6), thus suggesting that conversion to β-strand conformation observed in the presence of LTA by circular dichroism spectroscopy is representative of amyloid fibrils not responsive to ThT under the experimental conditions tested.

**Figure 7:**
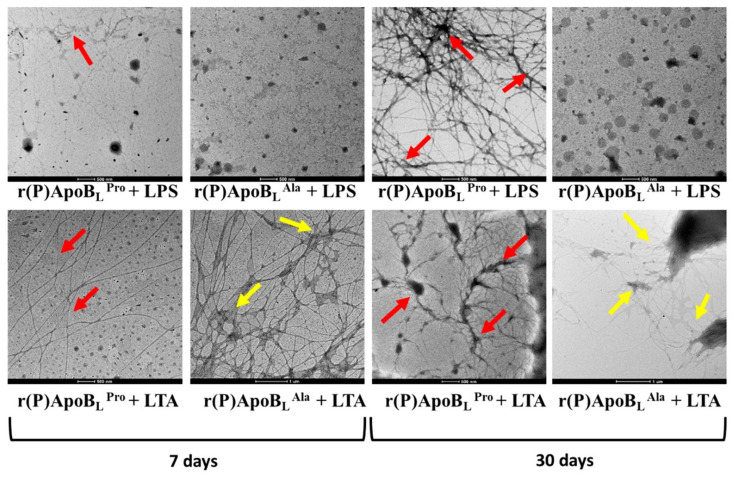
Transmission electron microscopy images of r(P)ApoB_L_^Pro^ and r(P)ApoB_L_^Ala^ aggregates obtained upon 7 and 30 days of incubation with LPS (**top**) or LTA (**bottom**) in 20 mM Sodium Phosphate at pH 7.4.

In order to further investigate the ability of ApoB-derived peptides to form amyloid-like fibrils upon incubation with bacterial surface components, peptides’ transmission electron microscopy analyses have been carried out after either 7 days or 30 days of incubation with LPS or LTA. Mature amyloid fibrils were detected in the case of r(P)ApoB_L_^Pro^ upon incubation with LPS, with more evident fibrils obtained upon 30 days incubation (red arrows in Figure 7). These data are in agreement with circular dichroism and ThT fluorescence emission analyses described above. When r(P)ApoB_L_^Pro^ peptide was incubated in the presence of LTA, mature fibrils were also evidenced by TEM analyses after both 7 days and 30 days incubation (Figure 7), in agreement with circular dichroism spectra analyses that evidenced a conversion to a prevalently β-sheet conformation upon incubation with LTA. However, it has to be highlighted that no increase of ThT fluorescence emission was, instead, detected when r(P)ApoB_L_^Pro^ was incubated with LTA probably because of the heterogeneity of peptide self-assembly induced by the interaction with LTA that might promote the formation of fibrils not responsive to ThT, as previously described for different amyloidogenic proteins in specific experimental conditions [61,62]. Similarly, in the case of r(P)ApoB_L_^Ala^, mature fibrils were detected by TEM only upon incubation with LTA, with an evident increase of fibrils branching after 30 days incubation (yellow arrows in the Figure 7). No fibrils were, instead, detected by TEM upon r(P)ApoB_L_^Ala^ incubation with LPS, although circular dichroism and ThT fluorescence emission analyses described above indicate the formation of β-sheet-rich structures. This behavior could be explained by considering that, differently from r(P)ApoB_L_^Pro^, a non-sigmoidal trend was obtained when r(P)ApoB_L_^Ala^ was incubated with LPS in the presence of ThT. Indeed, sigmoidal trends have been described for several known amyloid peptides [17,26,27,63]. It has to be highlighted that the molecular mechanism at the basis of thioflavin-T binding to an aromatic-hydrophobic groove, spanning across four consecutive beta-strands, provides a generic mode of recognition for amyloid dyes [64], but this recognition mode might not be strictly related to the presence of mature fibrils. Based on collected data, it might be hypothesized that, in the case of r(P)ApoB_L_^Ala^, the interaction with LPS induces a transition towards a β-sheet conformation with the consequent development of β-sheet-rich oligomers that might exert their toxic effects without giving rise to mature fibrils. Analyses of r(P)ApoB_L_^Pro^, r(P)ApoB_L_^Ala^, LPS or LTA alone in phosphate buffer are reported as negative controls (Supplementary Figure S7).

## 3. Discussion

The discovery and subsequent use of antibiotics more than 60 years ago undoubtedly changed the course of human history by allowing to cure previously deadly diseases. However, over several decades, an abundance of multidrug resistant bacteria has emerged, what makes most of the available antimicrobials largely ineffective. On the other hand, the discovery, development, manufacture and marketing of new antibiotics has significantly slowed down in the past 20 years [1,2,3]. In this scenario, naturally occurring host defense peptides (HDPs) are gaining great attention. Indeed, they are able to prevent infections in many organisms, a feature that opens interesting perspectives to the applicability of these peptides as a new class of antimicrobials and as a promising effective alternative to conventional antibiotics. They initially attracted attention solely for their direct antimicrobial activity. Indeed, many HDPs are characterized by a rapid action, and a broad spectrum of activity against Gram-positive and Gram-negative bacteria, viruses, fungi and parasites [7,65]. It is also increasingly emerging that many HDPs are endowed with additional biological roles, such as immunomodulatory, angiogenic, wound healing, anticancer and anti-biofilm activities. This has greatly increased the interest *versus* this class of molecules. HDPs often cause the death of microorganisms by interacting with the lipid bilayer and then determining membrane disruption in a specific, but not receptor-mediated, process [15,66]; several models have been proposed to describe this process of membrane permeabilization. In recent years, it has been also demonstrated that several HDPs have the ability to form β-sheet-rich amyloid-like fibrillar structures, what has led to postulate the existence of two seemingly unrelated classes of polypeptides, HDPs and fibril-forming (amyloidogenic) antimicrobial peptides, that share common mechanisms of cytotoxicity based on membrane disruption [67,68,69,70,71,72,73,74,75,76,77,78,79]. The deposits known as amyloids exhibit a characteristic cross-β structure that allows them to exert toxic effects by forming ion channels in bacterial cell membranes, thus causing membrane depolarization, energy drainage, and in some cases apoptosis [60]. It is emerging that these structures could play a key role also in the molecular mechanism underlying HDPs antimicrobial activity. Indeed, human HDPs generally kill invading microbes through a channel-forming mechanism. Several antimicrobial peptides have been found to be able to give rise to β-sheet structures, similar to those formed by amyloid peptides. Examples are represented by serum amyloid A, microcin E492, protegrin-1 (PG-1), temporins and lysozyme [70]. The analysis of the relationship between antimicrobial activity and amyloid formation will potentially enable significant advances in the comprehension of both amyloid diseases and the mechanism of action of bioactive antimicrobial peptides, thus paving the way for the generation of novel biocompatible materials suitable for diagnostic and/or therapeutic applications [71].

In the last years, our research group developed and optimized a bioinformatic tool that allows the precise and reliable localization of HDP-like sequences hidden in precursor human HDP releasing proteins [47]. A cryptic HDP has been identified in human apolipoprotein B (ApoB) and two recombinant versions of the identified sequence have been produced, a longer version, r(P)ApoB_L_, and a shorter version, r(P)ApoB_S_. P refers to a Pro residue present at the N-terminal of recombinant peptides because of the excision method used where peptides are released after the acidic cleavage of an Asp-Pro bond [47]. Peptides were demonstrated to be endowed with a broad-range antimicrobial activity, being effective on both Gram-negative and Gram-positive bacterial strains, with a notable anti-biofilm activity and with the ability to synergistically act in combination with EDTA or conventional antibiotics [9]. Peptides have been also found to display immunomodulatory properties, to target sensitive bacteria by depolarizing their cytoplasmic membrane, to show excellent cytotoxicity profiles, and not to select for bacterial resistance mechanisms [10,11,37,49].

In the present study, we demonstrate for the first time the ability of ApoB-derived peptides to form amyloid fibrils when in contact with components of bacterial surfaces. We also describe for the first time the impact of a single point mutation (7-Pro → 7-Ala) on the biological properties and on the molecular mechanism of the antibacterial activity of r(P)ApoB_L_ peptide. The obtained peptides containing Pro or Ala in position 7 have been here named r(P)ApoB_L_^Pro^ and r(P)ApoB_L_^Ala^, respectively.

Firstly, we performed a comparison between antimicrobial and anti-biofilm properties of the two versions of the peptide and found similar behaviors (Table 2, Figure 1). However, it has to be noticed that a significant difference between the two peptides was evidence when their bactericidal properties were evaluated towards *S. aureus* MRSA WKZ-2 and *P. aeruginosa* PAO1 bacterial cells, selected as a prototype of Gram-positive and Gram-negative bacteria, respectively. Results of colony counting assays revealed that only r(P)ApoB_L_^Ala^ exerts a strong bactericidal effect upon 24 h incubation (Figure 2). Indeed, in the case of r(P)ApoB_L_^Pro^ peptide, obtained data appear to support a bacteriostatic effect under the experimental conditions tested (Figure 2). This appears in agreement with data reported in the literature indicating that proline-to-alanine substitutions alter the stability, refolding process, and biological activity of ribonuclease onconase [72]. However, it has to be highlighted that the antimicrobial activity of both versions of r(P)ApoB_L_ peptide was found to be strongly influenced by the presence in solution of specific components of bacterial membranes or cell walls, such as LPS or LTA (Figure 2). Indeed, when peptides have been pre-incubated with LPS or LTA, their antimicrobial activity has been found to be significantly reduced, thus indicating that free LPS and LTA compete with molecules exposed on bacterial surfaces for the binding to peptide molecules. This strongly indicates that peptides antimicrobial activity is dependent from peptides interaction with LPS and LTA, as previously demonstrated for r(P)ApoB_L_^Pro^ peptide and LPS by ITC experiments [9,10], and as here indirectly demonstrated by circular dichroism conformational analyses of the peptides upon incubation with LPS or LTA. This is also in agreement with reported findings indicating that human serum lipoproteins, such as ApoB100, ApoA1 and ApoA2, are able to bind to LTA [73]. However, only in the case of ApoB100 and of the corresponding LDL, this interaction has a biological relevance by inhibiting LTA-induced cytokine releases from human and murine immune cells [73]. In the literature, evidence also exists about the correlation between the binding of HDPs to LPS molecules and cell agglutination process leading to cells death and lysis in the case of Gram-negative bacteria [19]. To deepen on the molecular events underlying peptides antimicrobial activity, we also analysed their ability to interact with a double lipid layer by differential scanning calorimetry. Obtained results did not highlight significant differences, since, in the case of both ApoB-derived peptides, a surface interaction with lipid head groups coupled with a lipid segregation process has been demonstrated, while both peptides are not able to destabilize the hydrophobic core of the bilayer (Figure 3). It has been reported that the formation of lipid domains in membranes can have a deep impact on the biological activity of the cells [74], such as an alteration of the diffusion rates of lipids and membrane proteins and an interference with membrane physiological curvature, with a consequent negative impact on key processes, such as cell division [14]. In the present manuscript, circular dichroism analyses reveal conformation shifts from a random-coil structure to a structural ensemble comprising significant β-sheet conformation, in the case of both peptides upon incubation with LPS or LTA (Figure 4c,d, Tables S3 and S4). Although incubation with LPS has a similar effect on both ApoB-derived peptides, that tend to assume a β-sheet-rich conformation, it has to be highlighted that, in the case of r(P)ApoB_L_^Ala^_,_ effects on conformation are registered at LPSs concentrations significantly lower than those necessary to have the same effect on r(P)ApoB_L_^Pro^ peptide, thus suggesting that peptide version with Ala in position 7 is characterized by a higher affinity for LPS molecules. No differences were, instead, revealed when peptides conformations were analysed in the presence of membrane-mimicking agents, such as TFE and SDS, that induce a shift of peptides secondary structure towards an α-helical conformation (Figure 4a,b; Supplementary Figure S1a,b), in agreement with data previously reported for r(P)ApoB_L_^Pro^ [9], and for several antimicrobial peptides [9,12,75].

Based on obtained results, ApoB-derived peptides antimicrobial activity appears also associated with the formation of β-sheet-rich amyloid-like structures. To support this hypothesis, experiments have been here performed to verify the ability of ApoB-derived peptides to form amyloid fibrils in vitro by Thioflavin T (ThT) fluorescence assays. An evident increase of ThT fluorescence emission, indicative of the formation of β-sheet-rich amyloid-like structures, was observed upon incubation of each peptide with LPS, with a higher fluorescence emission in the case of r(P)ApoB_L_^Pro^ (Figure 6). This evidence was confirmed by transmission electron microscopy analyses that revealed the formation of mature and branched fibrils (Figure 7). It has to be noted that no increase of ThT emission fluorescence was observed in the case of incubation with LTA, although the presence of fibrils was detected by electron microscopy probably because of the heterogeneity of peptide self-assembly induced by the interaction with LTA that might promote the formation of fibrils not responsive to ThT, as previously described for different amyloidogenic proteins in specific experimental conditions [61,62]. It has also to be highlighted that, in the case of r(P)ApoB_L_^Ala^ peptide, no fibrils have been detected by transmission electron microscopy upon incubation with LPS, even if circular dichroism analyses reveal a conversion from random coil to a β-sheet-rich conformation, thus suggesting that the presence of an Ala residue in position 7 might be responsible for a different mode of action against Gram-negative bacteria. Formation of amyloid-like structures playing a key role in peptides antimicrobial activity has been already described for different antimicrobial peptides, such as Uperin 3.5, a HDP isolated from the skin secretions of the Australian toadlet *Uperoleia mjobergii* [75]. This peptide has been demonstrated to rapidly form amyloid-like fibrils through a process that involves an α-helical intermediate that promotes the formation of fibrils sharing cytotoxic properties with amyloid species derived from peptides and proteins responsible for neurodegenerative diseases [76]. Indeed, data collected in the literature suggest a key cytotoxic role played by both prefibrillar aggregates and mature fibrils [70,77,78,79,80,81,82,83,84,85]. In the case of ApoB-derived peptides, mature fibrils were found not to exert antimicrobial effects on the bacterial strains under study (data not shown), in agreement with findings reported in the literature indicating that the oligomeric forms of amyloids are responsible for their cytotoxicity via membrane permeation, while their fibrillar conformations interact with the innate immune system to induce inflammation [86]. Based on obtained data, a correlation between the broad-spectrum antimicrobial activity of ApoB-derived peptides and their ability to form amyloid-like structures can be hypothesized. This is in agreement with recent data about the antimicrobial properties of amyloidogenic proteins responsible for the onset of severe diseases. As an example, it has been demonstrated that the overexpression of amyloidogenic Aβ peptide, responsible for the formation of plaques in the brain of Alzheimer disease patients, confers increased resistance to bacterial and viral infections [33].

Altogether, obtained data add an important tassel to the elucidation of ApoB-derived peptides mechanism of action and to the future design of peptides with additional properties that might be employed in the generation of novel anti-infective agents able to counteract drug-resistance phenotype.

## 4. Conclusions

In the present manuscript, we evaluated for the first time the effects of a single point mutation (Pro → Ala in position 7) on the biological properties of ApoB-derived peptide r(P)ApoB_L_^Pro^, a host defense peptide characterized by significant antimicrobial, anti-biofilm, immunomodulatory and wound healing properties, and by the ability to synergistically act in combination with EDTA or conventional antibiotics, while being neither toxic nor haemolytic towards eukaryotic cells. The two peptides have been found to share similar antimicrobial and anti-biofilm properties, and to interact with synthetic lipid bilayers simulating bacterial membranes in a similar fashion. However, this single point mutation appears to alter the efficiency of peptide direct killing, since r(P)ApoB_L_^Ala^ peptide exerts bactericidal effects in the same experimental conditions in which r(P)ApoB_L_^Pro^ exerts bacteriostatic effects. The molecular bases of this phenomenon need to be further clarified.

Furthermore, the antimicrobial activity of both versions of the peptide appears to be strictly dependent from their interaction with specific components of bacterial surfaces, such as LPS or LTA, which induce peptides conformational transition from random coil to β-sheet-rich amyloid-like structures. However, it has to be highlighted that r(P)ApoB_L_^Ala^ peptide tends to assume a prevalently β-structure conformation in the presence of LPS concentrations significantly lower than those required to have the same effect on r(P)ApoB_L_^Pro^, thus suggesting that the presence of Ala in position 7 is responsible for a higher affinity for LPS. ThT fluorescence assays and transmission electron microscopy analyses indicate that the incubation of peptides with LPS or LTA induces the formation β-sheet-rich amyloid-like fibrils. An exception is represented by r(P)ApoB_L_^Ala^ upon incubation with LTA. Indeed, in this case, conformational transition towards β-sheet structures is not accompanied by the formation of mature fibrils, what might be indicative of a different mechanism adopted by this peptide to exert antimicrobial effects on Gram-positive bacteria.

In conclusion, analysed single point mutation seems to have effects on the molecular mechanisms underlying peptide antimicrobial activity even if, in the case of both versions of the peptide, a correlation appears between self-assembling state and antibacterial properties. Altogether, these findings add an important tassel to the elucidation of the molecular mechanisms at the basis of peptides mechanism of action.

## 5. Materials and Methods

### 5.1. Materials

All the reagents were purchase from Sigma-Merck (Milan, Italy), unless specified otherwise.

### 5.2. Production of Recombinant Peptides

Primer directed PCR mutagenesis was used to introduce the Pro7Ala single point mutation (QuikChange II Site-Directed Mutagenesis Kit, Agilent, Santa Clara, CA, USA). Site-directed mutagenesis was verified by dideoxy automated fluorescent sequencing. Expression of recombinant peptides was carried out as previously described [48,86] and the obtained chimeric proteins were purified by nickel affinity chromatography as previously described [48]. In each case, the peptide was released from the carrier onconase by hydrolysis in acidic conditions. Since the carrier is insoluble at neutral or alkaline pH, the peptide was isolated from insoluble components by repeated cycles of centrifugation. A final gel-filtration step was added, in order to remove salts used along the purification process and that tend to attach to the peptides. Pure peptides were finally lyophilized, dissolved in pure water and quantified by BCA assay (Thermo Fisher Scientific, Waltham, MA, USA).

### 5.3. Liposomes Preparation

The lipids 1,2-dipalmitoyl-sn-glycero-3-phosphoethanolamine (DPPE) and 1,2-dipalmitoyl-sn-glycero-3-phospho-1′-rac-glycerol (DPPG) were purchased from Avanti Polar Lipids Inc. (Alabaster, AL, USA) and used without further purifications. Appropriate amounts of lipids were weighted in a glass vial and dissolved in the organic solvent chloroform. A thin film was produced by evaporating the organic solvent by gentle dry nitrogen gas. Samples were placed in a vacuum overnight, to remove the final traces of the solvent. The dried lipids were then hydrated in the liquid-crystalline phase at the temperature of 70 °C with an appropriate amount of 20 mM phosphate buffer at pH 7.4, and vigorously vortexed to obtain a suspension of multilamellar vesicles (MLVs). The obtained liposomes were composed of DPPE/DPPG 8:2 (mol/mol).

### 5.4. Differential Scanning Calorimetry (DSC)

DSC experiments were performed by means of a nano-DSC from TA Instruments (New Castle, DE, USA). MLVs were used for all DSC experiments since they provide the better resolution of the peak. Briefly, a volume of 300 μL of 0.5 mM vesicles suspension of DPPE/DPPG 8:2 (mol/mol) in the absence or in the presence of peptides was placed in the calorimetry vessel, and successive heating and cooling scans were obtained at 1 °C/min over the temperature range of 25–70 °C. The excess heat capacity function (<ΔCp>) was obtained after baseline subtraction. A buffer-buffer scan was subtracted from the sample scans. The samples composed by lipid suspension and peptide under test were prepared immediately before the DSC experiments by adding the appropriate amount of peptide to the preformed lipid suspension at room temperature. The obtained data were analysed by means of NanoAnalyze software and plotted by using the Origin software package (OriginLab, Northampton, MA, USA). Any changes in DSC profiles were attributed to the effect of the bound peptide on the membranes, in agreement with previous findings indicating that non-interacting peptide molecules do not affect membrane thermotropic properties.

### 5.5. Bacterial Strains and Growth Conditions

Six bacterial strains were used in the present study, i.e., *Escherichia coli* ATCC 35218, *Pseudomonas aeruginosa PAO1*, *Bulkolderia cenocepacia* J2315, methicillin-resistant *Staphylococcus aureus* MRSA WKZ-2, *Bacillus subtlis subsp. spizizenii* ATCC 6633, and *Staphylococcus aureus* ATCC 12600. All the bacterial strains were grown in Muller Hinton Broth (MHB) or in Nutrient Broth (NB), both purchased from Becton Dickinson (Difco, Franklin Lakes, NJ, USA), and on Tryptic Soy Agar (TSA; Oxoid Ltd., Hampshire, UK). In all the experiments, bacteria were inoculated and grown overnight in MHB at 37 °C. The next day, bacteria were transferred to a fresh MHB or NB tube and grown to mid-logarithmic phase.

### 5.6. Antimicrobial Activity Assays

The antimicrobial activity of r(P)ApoB_L_^Pro^ and r(P)ApoB_L_^Ala^ peptides was tested towards all the bacterial strains described above. Antimicrobial activity assays were performed as previously described [9,12]. Following an overnight incubation of bacteria with peptides at desired concentrations, MIC_100_ values were determined as the lowest peptide concentration responsible for no visible bacterial growth.

### 5.7. Anti-Biofilm Activity

Anti-biofilm activity of r(P)ApoB_L_^Ala^ peptide was tested on *P. aeruginosa* PAO1 and *S. aureus* MRSA WKZ-2 bacterial strains. Experiments were performed as previously described [9,11,12,49]. Briefly, bacteria were grown overnight in MHB (Becton Dickinson Difco), and then diluted to 1 × 10^8^ CFU/mL in fresh 0.5× MHB containing increasing peptide concentrations (0–20 µM in the case of *P. aeruginosa* PAO1 and 0–2.5 µM in the case of *S. aureus* MRSA WKZ-2). Incubations with each peptide were carried out either for 4 h, in order to test peptide effects on biofilm attachment, or for 24 h, in order to test peptide effects on biofilm formation. When peptide effects on preformed biofilm were evaluated, bacterial biofilms were formed for 24 h at 37 °C, and then treated with increasing concentrations of the peptide. In all the cases, at the end of the incubation, anti-biofilm activity was tested by crystal violet assay as previously described [9]. To this purpose, samples optical absorbance values were determined at 630 nm by using a microtiter plate reader (FLUOstar Omega, BMG LABTECH, Ortenberg, Germany).

### 5.8. Circular Dichroism Spectroscopy

CD spectra of r(P)ApoB_L_^Pro^ and r(P)ApoB_L_^Ala^ peptides were recorded with a J-810 spectropolarimeter equipped with a Peltier temperature control system (Model PTC-423-S, Jasco Europe, Cremella, LC, Italy) as previously described [13]. Far-ultraviolet (Far-UV) measurements (198–260 nm) were carried out in 20 mM sodium phosphate pH 7.4 at 20 °C by using a 0.1 cm optical path length cell. Spectra were recorded with a time constant of 4 s, a 1 nm bandwidth, and a scan rate of 20 nm min^−1^. Spectra are reported in terms of mean residue ellipticity, calculated by dividing the total molar ellipticity by the number of amino acids in the molecule. Each spectrum was corrected by subtracting the background and reported without further signal processing. Lyophilized peptides were dissolved in ultra-pure water (Romil, Waterbeach, Cambridge, UK) at a concentration of 100 µM, determined on the basis of peptide dry weight and BCA assay (Thermo Fisher Scientific). CD spectra of the peptides were collected in the absence or in the presence of increasing concentrations of trifluoroethanol (TFE), sodium dodecyl sulphate (SDS), lipopolysaccharide (LPS) from *E. coli* 0111:B4 strain, or lipoteichoic acid (LTA) from *S. aureus*. CD spectra were corrected by subtracting every time the contribution of the compound under test at any given concentration. When the effects of bacterial cells on peptides’ conformations were analysed, prior to analyses, peptides (10 μM) were pre-incubated with *P. aeruginosa* PAO1 (1.25 × 10^5^) or *S. aureus* MRSA WKZ-2 (3.75 × 10^5^) bacterial cells. Raw spectra were corrected for cell contribution. Three acquisitions for each spectrum were recorded. Deconvolutions of CD spectra were obtained using the web-based program CDPRO (http://lamar.colostate.edu/~sreeram/CDPro/, accessed on 22 November 2019).

### 5.9. Killing Kinetic Studies

To kinetically analyse bacterial killing by ApoB-derived peptides, experiments were performed on *P. aeruginosa* PAO1 and on *S. aureus* MRSA WKZ-2 bacterial strains upon incubation with 10 µM r(P)ApoB_L_^Pro^ or r(P)ApoB_L_^Ala^, as previously described [9,10]. Peptides were assayed alone or upon pre-incubation with 0.1 mg/mL LPS or LTA.

### 5.10. In Situ Real-Time ThT Fluorescence Assays

To perform in situ real-time ThT fluorescence assays, recombinant peptides (10 μM) were incubated alone or in the presence of 0.1 mg/mL LPS or LTA in 20 mM sodium phosphate pH 7.4 (0–3600 min). All the samples were mixed with ThT (10 μM) and the fluorescence emission was acquired at 482 nm upon excitation at 450 nm by using a GloMax^®^ Discover System (Promega, Madison, WI, USA). In all the cases, three independent experiments were performed with internal triplicate determinations.

### 5.11. Statistical Analysis

Statistical analyses were performed by using a Student’s *t*-Test. Significant differences were indicated as * (*p <* 0.05), ** (*p <* 0.01), *** (*p <* 0.001), or **** (*p <* 0.0001).

### 5.12. Transmission Electron Microscopy

Transmission electron microscopy analyses have been carried out after either 7 days or 30 days of incubation for each peptide (10 µM) in the presence of 0.1 mg/mL of LPS or LTA in 20 mM sodium phosphate pH 7.4. Analyses of r(P)ApoB_L_^Pro^, r(P)ApoB_L_^Ala^, LPS or LTA alone have been carried out in phosphate buffer and reported as negative controls. At the end of the incubations, each sample has been deposited onto a carbon coated copper grid (200 mesh) and allowed to air dry before imaging. TEM images were acquired by using a FEI TECNAI G2 200 kV microscope and an acceleration voltage of 120 kV.

## Figures and Tables

**Table 1:** Physico-chemical properties of peptides deriving from human apolipoprotein B.

Peptide Name	Sequence	Molecular Weight	Net Charge at Neutral pH	Isoelectric Point	Grand Average of Hydropathicity
r(P)ApoB_L_^Pro^	PHVALKPGKLKFIIPSPKRPVKLLSGGNTLHLVSTTKT	4074.96 Da	7.2	11.43	0.005
r(P)ApoB_L_^Ala^	PHVALKAGKLKFIIPSPKRPVKLLSGGNTLHLVSTTKT	4048.92 Da	7.2	11.43	0.005

**Table 2:** Minimal inhibitory concentration (MIC_100_, µM) values determined for r(P)ApoB_L_^Pro^ and r(P) ApoB_L_^Ala^ tested on a panel of Gram-negative and Gram-positive planktonic bacterial cells.

Gram-Negative Strains	r(P)ApoB_L_^Pro^	r(P)ApoB_L_^Ala^
*Escherichia coli* ATCC 35218	2.5	2.5
*Pseudomonas aeruginosa* PAO1	5–10	2.5–10
*Bulkolderia cenocepacia* J2315	40	40
Gram-positive strains		
*Staphylococcus aureus* MRSA WKZ-2	5–10	2.5
*Bacillus subtlis subsp. spizizenii* ATCC 6633	5	5
*Staphylococcus aureus* ATCC 12600	20	20

**Table 3:** Thermodynamic parameters for the gel to liquid phase transition of DPPE/DPPG MLVs in the absence and in the presence of r(P)ApoB_L_^Pro^ and r(P)ApoB_L_^Ala^ peptides.

System	ΔH_m_ (kJ mol^−1^) ^a,b,c^	T_m_ (°C) ^b,d^
DPPE/ DPPG	39.7	60.5
+ r(P) ApoB_L_^Pro^	37.5	62.0
+ r(P) ApoB_L_^Ala^	34.8	62.3

^a^ Normalization against total moles of lipids. ^b^ The reported values refer to the equilibrium scans (second heating scans). ^c^ The errors are ±10% of the reported values. ^d^ The errors are ±0.2 °C of the reported values.

## Data Availability

Data is contained within the article and Supplementary Material.

## Supplementary Materials

The following are available online at https://www.mdpi.com/article/10.3390/ph14070631/s1, Figure S1: Far-UV CD spectra of r(P)ApoB_L_^Ala^ in the presence of increasing concentrations of membrane-mimicking agents TFE (a) and SDS (b). Dashed lines represent peptides in 20 mM sodium phosphate pH 7.4, Table S1: CD spectra deconvolution percentages of r(P)ApoB_L_^Pro^ and r(P)ApoB_L_^Ala^ peptides in 20 mM Sodium Phosphate pH 7.4 buffer and in presence of 50% TFE. Secondary structure percentages were calculated using CDPRO software, Table S2: CD spectra deconvolution percentages of r(P)ApoB_L_^Pro^ and r(P)ApoB_L_^Ala^ peptides in 20 mM Sodium Phosphate pH 7.4 and in presence of 40 mM SDS. Secondary structure percentages were calculated using CDPRO software, Figure S2: Far-UV CD spectra of r(P)ApoB_L_^Ala^ in presence of increasing concentration of LPS. Dashed line represents peptide in 20 mM Sodium Phosphate pH 7.4, Table S3: CD spectra deconvolution percentages of r(P)ApoB_L_^Pro^ and r(P)ApoB_L_^Ala^ peptides in 20 mM Sodium Phosphate pH 7.4 buffer and in presence of 0.2 mg/mL of LPS. Secondary structure percentages were calculated using CDPRO software, Table S4: CD spectra deconvolution percentages of r(P)ApoB_L_^Pro^ and r(P)ApoB_L_^Ala^ peptides in 20 mM Sodium Phosphate pH 7.4 buffer and in presence of 0.2 mg/mL LTA. Secondary structure percentages were calculated using CDPRO software, Figure S3: Raw Far-UV CD spectra of r(P)ApoB_L_^Pro^ and r(P)ApoB_L_^Ala^ in the presence of Gram-negative *P. aeruginosa* PAO1 (a) or in the presence of Gram-positive *S. aureus* MRSA WKZ-2 (b). Black lines represent spectra of bacterial cells after 270 min of incubation, Figure S4: Far-UV CD spectra of r(P)ApoB_L_^Ala^ (a) and r(P)ApoB_L_^Pro^ (b) upon incubation with Gram-negative *P. aeruginosa* PAO1 strain for different time intervals. Dashed lines represent peptides in 20 mM Sodium Phosphate pH 7.4, Figure S5: Far-UV CD spectra of r(P)ApoB_L_^Ala^ (a) and r(P)ApoB_L_^Pro^ (b) upon incubation with Gram-positive *S. aureus* MRSA WKZ-2 strain for different time intervals. Dashed lines represent peptides in 20 mM Sodium Phosphate pH 7.4, Figure S6: In situ real-time ThT fluorescence assays of r(P)ApoB_L_^Pro^ and r(P)ApoB_L_^Ala^ peptides upon incubation with LTA. In all the cases, reported data derive from three independent experiments with internal triplicate determinations, Figure S7: Transmission electron microscopy images of r(P)ApoB_L_^Pro^, r(P)ApoB_L_^Ala^, LPS and LTA upon 7- and 30-days incubation in 20 mM Sodium Phosphate pH 7.4.
