# Supplementary material for: Impact of a Single Point Mutation on the Antimicrobial and Fibrillogenic Properties of Cryptides from Human Apolipoprotein B
Source: Pharmaceuticals (Basel). 2021 Jun 29;14(7):631. doi: 10.3390/ph14070631 (PMC8308739; doi:10.3390/ph14070631)

Supplementary Figure S1. Far-UV CD spectra of r(P)ApoB$_L^{Ala}$ in the presence of increasing concentrations of membrane-mimicking agents TFE (a) and SDS (b). Dashed lines represent peptides in 20 mM sodium phosphate pH 7.4.

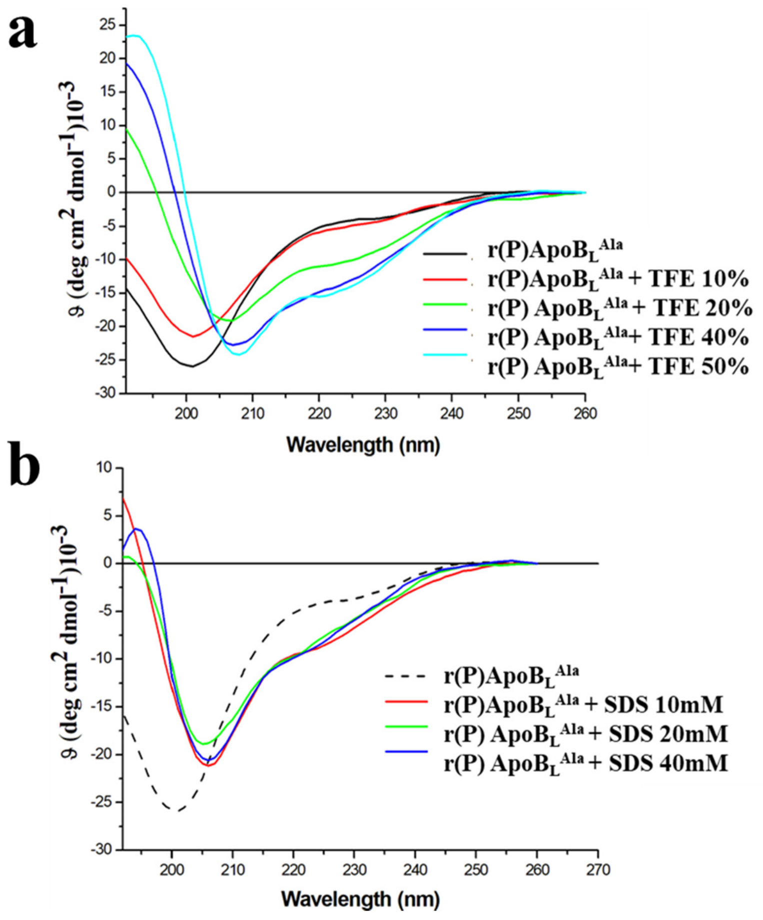

Supplementary Table S1. CD spectra deconvolution percentages of r(P)ApoB$_L^{Pro}$ and r(P)ApoB$_L^{Ala}$ peptides in 20 mM Sodium Phosphate pH 7.4 buffer and in presence of 50% TFE. Secondary structure percentages were calculated using CDPRO software.

	**% α-helix**	**% β- sheet**	**% random coil**
**r(P)ApoB$_L^{Pro}$**	**20.7**	**7.8**	**71.7**
**r(P)ApoB$_L^{Ala}$**	**26.4**	**9**	**64.7**
**r(P)ApoB$_L^{Pro}$ + 50% TFE**	**70.4**	**1.9**	**27.5**
**r(P)ApoB$_L^{Ala}$ + 50% TFE**	**62.1**	**3.2**	**34.6**

Supplementary Table S2. CD spectra deconvolution percentages of r(P)ApoB$_L^{Pro}$ and r(P)ApoB$_L^{Ala}$ peptides in 20 mM Sodium Phosphate pH 7.4 and in presence of 40 mM SDS. Secondary structure percentages were calculated using CDPRO software.

	**% α-helix**	**% β- sheet**	**% random coil**
**r(P)ApoB$_L^{Pro}$**	**20.7**	**7.8**	**71.7**
**r(P)ApoB$_L^{Ala}$**	**26.4**	**9**	**64.7**
**r(P)ApoB$_L^{Pro}$ + 40 mM SDS**	**41.6**	**6.6**	**51.7**
**r(P)ApoB$_L^{Ala}$ + 40 mM SDS**	**44.4**	**5.8**	**49.7**

Supplementary Figure S2. Far-UV CD spectra of r(P)ApoB$_L^{Ala}$ in presence of increasing concentration of LPS. Dashed line represents peptide in 20 mM Sodium Phosphate pH 7.4.

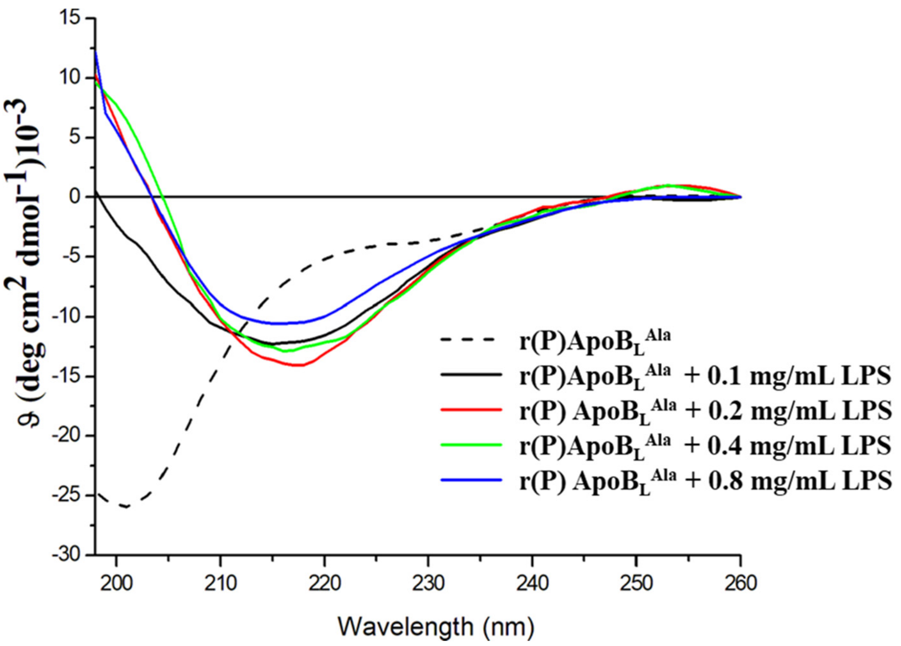

Supplementary Table S3. CD spectra deconvolution percentages of r(P)ApoB$_L^{Pro}$ and r(P)ApoB$_L^{Ala}$ peptides in 20 mM Sodium Phosphate pH 7.4 buffer and in presence of 0.2 mg/mL of LPS. Secondary structure percentages were calculated using CDPRO software.

	% α-helix	% β- sheet	% random coil
**r(P)ApoB$_L^{Pro}$**	**20.7**	**7.8**	**71.7**
**r(P)ApoB$_L^{Ala}$**	**26.4**	**9**	**64.7**
**r(P)ApoB$_L^{Pro}$ + 0.2 mg/mL LPS**	**22.8**	**18.5**	**58.8**
**r(P)ApoB$_L^{Ala}$ + 0.2 mg/mL LPS**	**19**	**35.6**	**45.3**

Supplementary Table S4. CD spectra deconvolution percentages of r(P)ApoB$_L^{Pro}$ and r(P)ApoB$_L^{Ala}$ peptides in 20 mM Sodium Phosphate pH 7.4 buffer and in presence of 0.2 mg/mL LTA. Secondary structure percentages were calculated using CDPRO software.

	% α-helix	% β- sheet	% random coil
**r(P)ApoB$_L^{Pro}$**	**20.7**	**7.8**	**71.7**
**r(P)ApoB$_L^{Ala}$**	**26.4**	**9**	**64.7**
**r(P)ApoB$_L^{Pro}$ + 0.2 mg/mL LTA**	**17.2**	**30.6**	**52.2**
**r(P)ApoB$_L^{Ala}$ + 0.2 mg/mL LTA**	**21.8**	**14.6**	**63.6**

Supplementary Figure S3. Raw Far-UV CD spectra of r(P)ApoB$_L^{Pro}$ and r(P)ApoB$_L^{Ala}$ in the presence of Gram-negative *P. aeruginosa* PAO1 (a) or in the presence of Gram-positive *S. aureus* MRSA WKZ-2 (b). Black lines represent spectra of bacterial cells after 270 min of incubation.

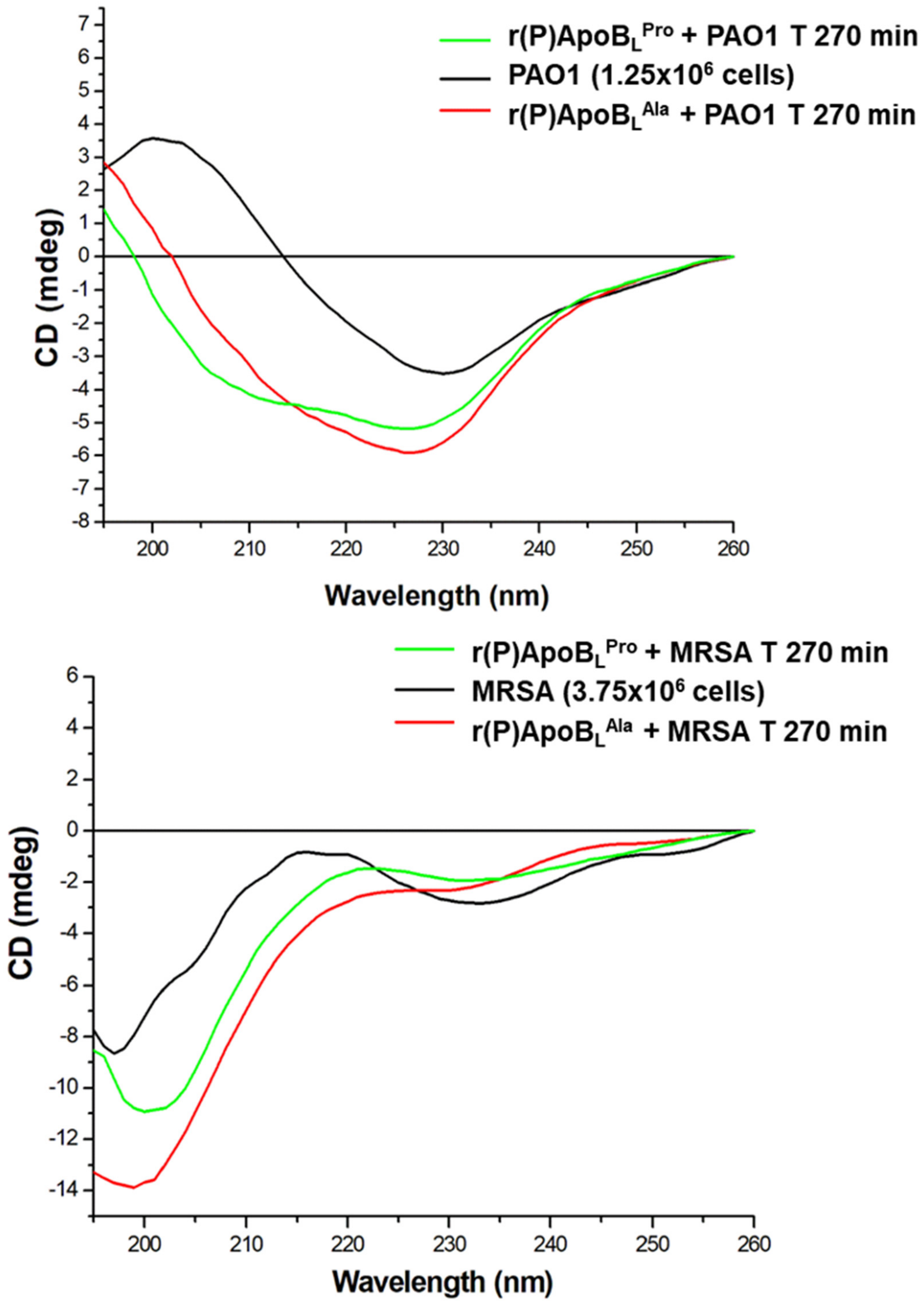

Supplementary Figure S4. Far-UV CD spectra of r(P)ApoB$_L^{Ala}$ (**a**) and r(P)ApoB$_L^{Pro}$ (**b**) upon incubation with Gram-negative *P. aeruginosa* PAO1 strain for different time intervals. Dashed lines represent peptides in 20 mM Sodium Phosphate pH 7.4.

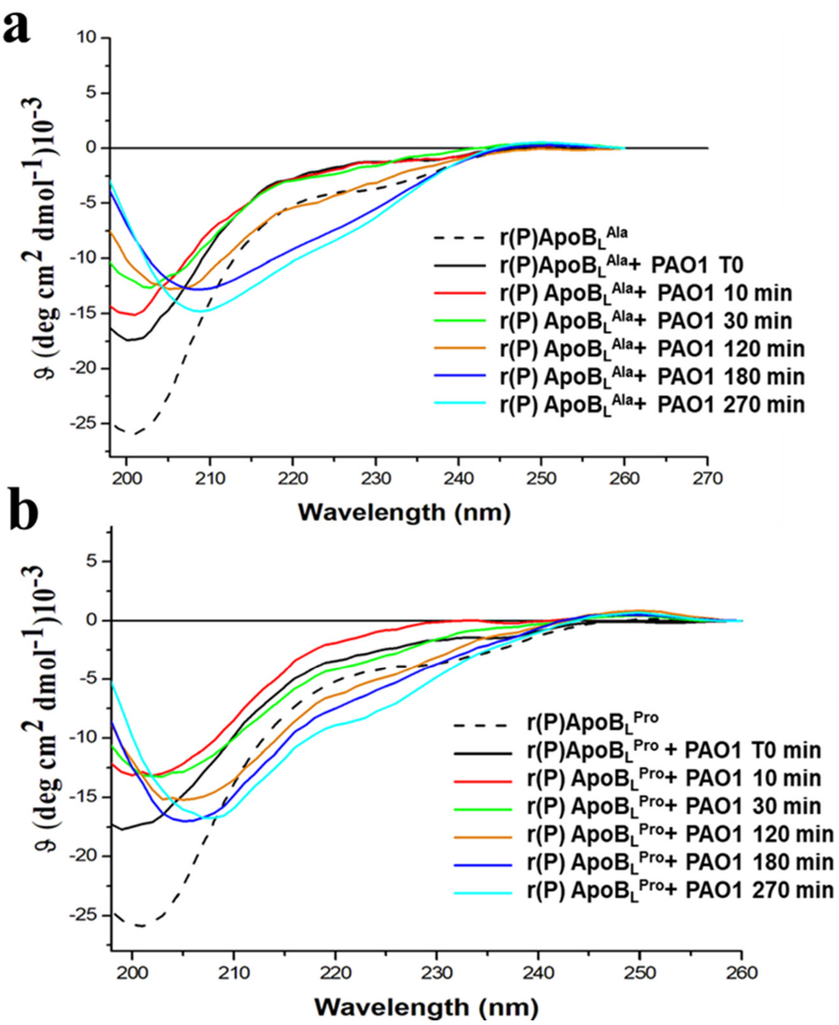

Supplementary Figure S5. Far-UV CD spectra of r(P)ApoB$_L^{Ala}$ (**a**) and r(P)ApoB$_L^{Pro}$ (**b**) upon incubation with Gram-positive *S. aureus* MRSA WKZ-2 strain for different time intervals. Dashed lines represent peptides in 20 mM Sodium Phosphate pH 7.4.

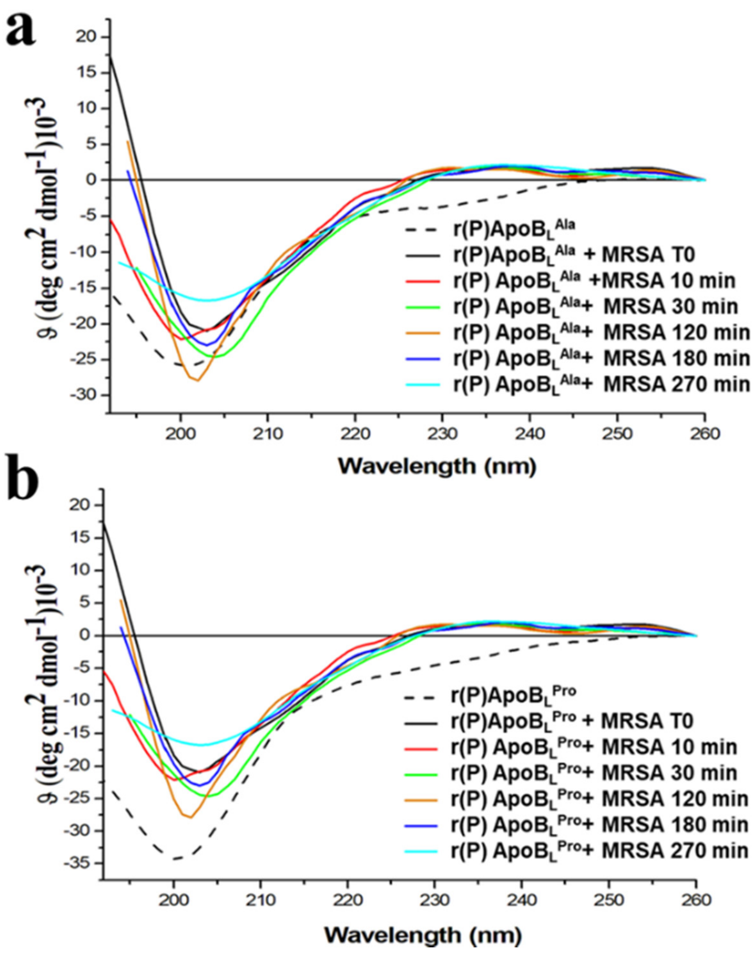

Supplementary Figure S6. *In situ* real-time ThT fluorescence assays of r(P)ApoB$_L^{Pro}$ and r(P)ApoB$_L^{Ala}$ peptides upon incubation with LTA. In all the cases, reported data derive from three independent experiments with internal triplicate determinations.

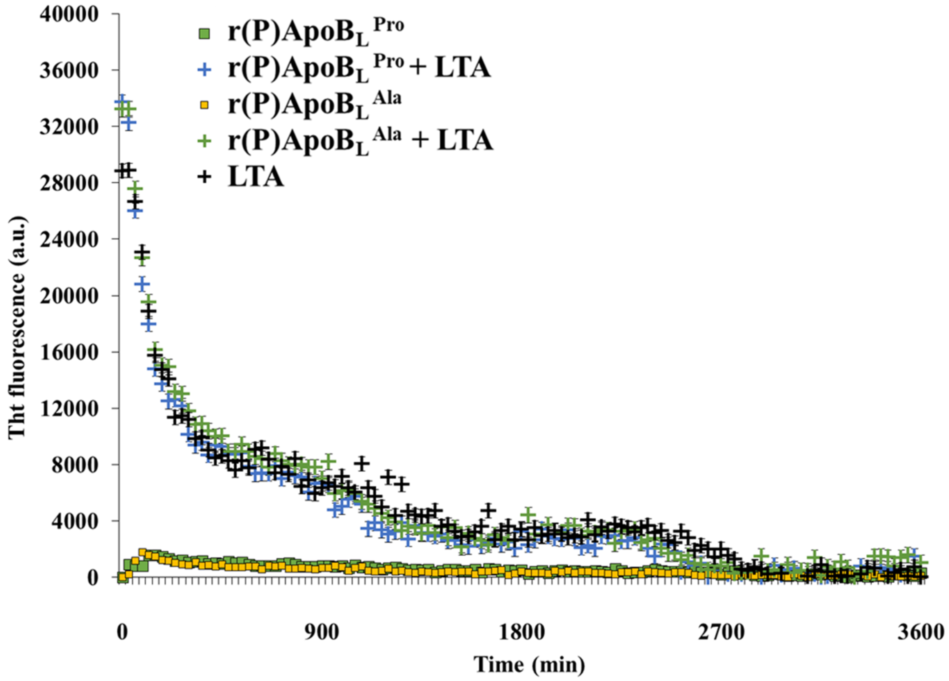

Supplementary Figure S7. Transmission electron microscopy images of r(P)ApoB$_L^{Pro}$, r(P)ApoB$_L^{Ala}$, LPS and LTA upon 7- and 30-days incubation in 20 mM Sodium Phosphate pH 7.4.

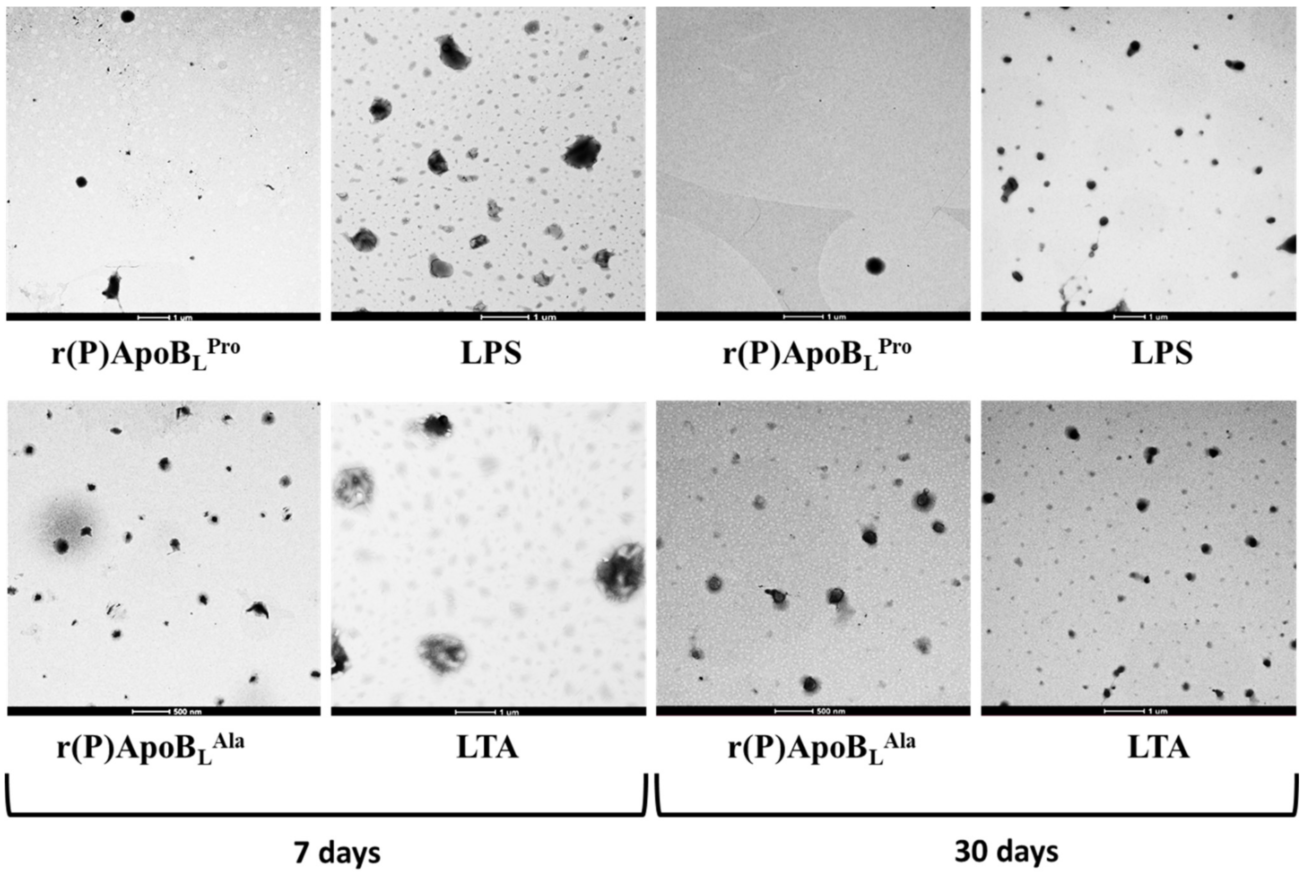

Supplement: Supplementary file 1 [file pharmaceuticals-14-00631-s001.zip › pharmaceuticals-1267485-supplementary.pdf]
